# Optimizing SRS for Brain Metastases: Understanding the Volume–Gradient Index Relationship

**DOI:** 10.3390/jcm14228123

**Published:** 2025-11-17

**Authors:** Tia Popescu-Bârhală, Ionuţ Dumitru, Mihai-Ştefan Bârhală, Horia-Dan Lişcu

**Affiliations:** 1 Faculty of Physics, University of Bucharest, Atomiștilor Street 405, 077125 Măgurele, Romania; mihai.barhala@gmail.com; 2 Neolife Bucharest, Ficusului 40 Boulevard, 013975 Bucharest, Romania; ionut94@gmail.com; 3 Department of Oncological Radiotherapy and Medical Imaging, “Carol Davila” University of Medicine and Pharmacy, Eroii Sanitari 8 Boulevard, 050474 Bucharest, Romania

**Keywords:** stereotactic radiotherapy, quality metrics, treatment planning, evaluation

## Abstract

**Background/Objectives**: The goal is to provide a straightforward framework for generating SRS-SRT plans that reliably meet high-quality dosimetric standards. That posed some questions such as which plan quality metrics should be utilized to evaluate a plan, what influences plan quality metrics the most, and finally, how to best optimize plan geometry. Our work has primarily concentrated on the second question, guided by our clinical experience. **Methods**: A dataset for statistical analysis was compiled by retrospectively reviewing 200 individual SRS-SRT target volumes from two centers. From the gathered data, several Linear Regression models were generated to assess the variability of plan quality metrics using statistical analysis. The most important regressor in the models were revealed to be target volume (TV), followed by a flag type variable that indicates whether the plan used to treat the referenced target contained multiple targets (MT) or not. **Results**: Every doubling of TV lowers Gradient Index (GI) sharply (−0.55 to −0.17) while Gradient Measure (GM) increases moderately (+0.024 cm to +0.07 cm). The model explains 85% of the variation in GI (R^2^ = 0.85) and 84% of GM. **Conclusions**: In small lesions, GI seems to be a more sensitive evaluation metric for sub-CC SRS targets, compared to GM. Dose per fraction appeared to have had no significant effect. Treating multiple targets in the same plan appears to add an average of +0.19 to GI, independent of volume, while for GM by +0.027 cm.

## 1. Introduction

Stereotactic radiotherapy as a high dose per fraction treatment modality has been an emerging and quickly developing field of study for the past years. All three—stereotactic radiosurgery (SRS), stereotactic radiation therapy (SRT), and stereotactic body radiation therapy (SBR)—refer to the highly precise delivery of radiation enabled by advanced technology [1,2]. In our setting, the technological capability includes a Varian TrueBeam equipped with HD Multi-leaf Collimator (HD-MLC), six degree-of-freedom couch, and IDENTIFY surface guided system (SGRT) working in conjunction with a GE Discovery Computed Tomography (CT), equipped with Varian Respiratory Gating for Scanners (RGSC) utilized as a simulator. This allows us to simulate and treat both SRS and SBRT cases within our clinic while utilizing advanced scanning techniques such as 4D-CT, Deep Inspiration Breath Hold (DIBH), and End Expiration Breath Hold (EEBH) while utilizing gating and intra-fraction motion management during treatment [3].

In order to safely standardize and improve our treatment planning processes, we evaluated the plan quality metrics first introduced by Paddick [4]. Paddick’s work provides a conceptual framework for gradient metrics, but a practical gap exists in their direct operational use for routine quality assurance. The specific practical need and the motivation for this study was to develop an institutional, predictive QA model based on our own standardized planning conditions. The goal was to move beyond a simple pass/fail check and establish expectable and achievable gradient values based on key plan characteristics, most notably target volume. This work is not intended to re-validate the known physical relationships but rather to provide an operational contribution. However, after further literature review [5,6] more studies were required to investigate the two different metrics, Gradient Index (GI) and Gradient Measure (GM), in order to decide which to use as a plan differentiator when assessing dose gradients within treatment plans. GI is defined as the ratio of the volume of 50% isodose to the volume of reference isodose, while GM is a three-dimensional evaluation factor of the distance to the 50% isodose from the 100% prescription dose [7].

A dataset was compiled to investigate the plan quality metrics that we use clinically in our department (Table 1) [5,8].

## 2. Materials and Methods

**Figure 1:**
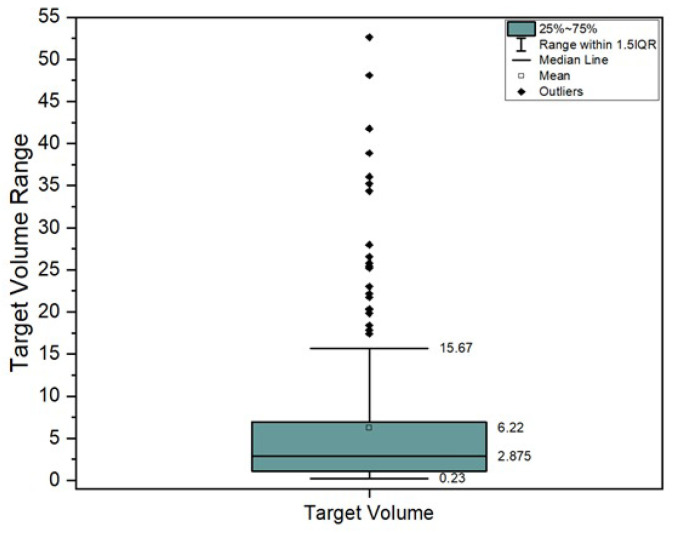
Descriptive statistics of the target volumes.

A cohort of 200 SRS-SRT individual target volumes from 104 patients were analyzed. The inclusion criteria for patient selection consisted in cerebral SRS-SRT treated lesions between 2021 and 2025 across two Neolife radiotherapy centers in Bucharest, Romania. We excluded lesions larger than 60 cc and those which did not fully complete the treatment course. Descriptive statistics can be found in Figure 1.

The patients were simulated using a GE Discovery CT (GE HealthCare, Chicago, IL, USA) with slice thickness of 1.25 mm for all SRT patients. Encompass SRS Fibreplast system (Q-Fix, Palmyra, PA, USA) was utilized for the simulation together with a Universal Couchtop Type-S and an SRS adaptor from CQ Medical (Avondale, PA, USA). Contouring and treatment planning was performed by different personnel (physician and physicist, respectively) and was subjected to a second verification by a second physicist as per internal protocol. Since our two departments follow the same training process for all medical physicists performing the treatment plans and the same protocols for simulation, the results obtained have minimal variability. The Gross Tumor Volume (GTV) contours were defined on a post-contrast T1-weighted high resolution Magnetic Resonance Imaging (MRI) and copied over to the rigid co-registered planning CT. The Planning Target Volumes (PTV’s) were defined as a 1–3 mm isotropic expansion of the GTVs. The dose distribution was calculated using both Anisotropic Analytical Algorithm (AAA) and Acuros XB dose calculation Algorithms version 16.1, with dose grid size of 1.25 mm, divided by the two centers. Patients were treated using Varian TrueBeam (Varian Medical Systems, Inc., Palo Alto, CA, USA) with HD-MLC.

Using the gathered data, two linear regression models with GI and GM as the response variables were generated in order to ascertain the relevant regressors and coefficients that may help explain their variability. Table 2 contains the currently recorded variables in our dataset [9].

**Figure 2:**
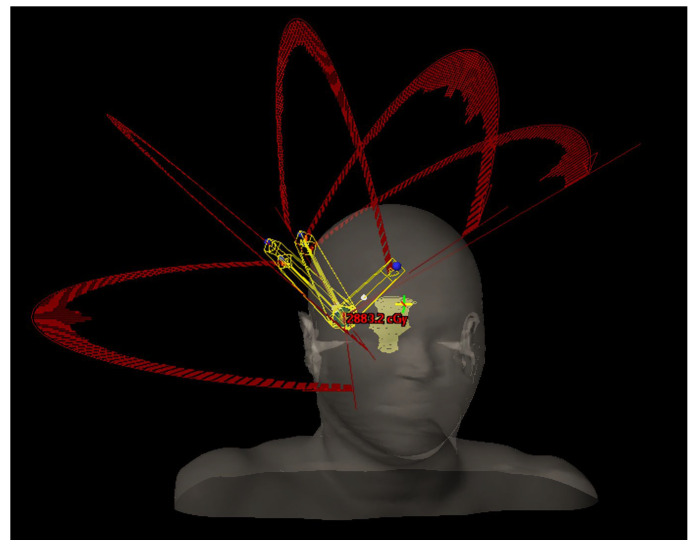
Generic SRS/SRT beam arrangement—5 partial arcs.

Regarding the geometrical arrangement of treatment arcs, the commonly approached technique is the following: set a partial arc or a full arc at 0° (±5°) couch angle, depending if the target is centered or not; set a partial arc at 90° or 270° (±5°), depending on the position of the target (for left hemisphere targets the chosen couch angle is 90° to prevent imager–couch collisions, and vice versa); set a minimum of 2 partial arcs, equally spaced, in the available range between the primary arcs (add extra arcs or double the existing arcs if the dose per fraction is increased over 15–20 Gy and space accordingly); adjust the length of the arcs in order to avoid direct beam entry through the eyes/ lenses; adjust the arc lengths (for targets in the posterior brain) to minimize low-dose exposure to healthy tissue [2]. In particular, if the target position and proximity to the skull are advantageous (e.g., upper part of parietal/ frontal lobe), an additional arc with the couch angle symmetric to the midline could be used. Another key consideration based on our clinical experience, adding an arc ensures the gantry speed remains above 1.5–2° per second at each control point, preventing significant mechanical variations during beam delivery. Such example of geometry is displayed in Figure 2.

For data collection, the above variables were recorded once the plan was finalized, reviewed by a second physicist, evaluated, and approved by the physician. The first step is normalizing so that at least 98% of the target volume receives at least 98% of the prescribed dose. Data required for dose metric calculations were extracted from the Dose Volume Histogram (DVH), either directly from the BODY and PTV structures or from an auxiliary structure encompassing the entire 50% isodose [10]. For plans with multiple lesions, only the latter approach (auxiliary structures) was used for evaluation on the plan summation, as it yields dose metrics for each individual lesion. The rest of the data were directly gathered from the plan parameters, plan structures, and so on. The only exceptions regarding the plan normalization were the targets that posed a threat of not respecting the clinical goals of the Planning Risk Volumes (PRVs). Those particular plans were normalized to the highest coverage possible while still maintaining the clinical goals agreed upon with the physician.

Besides the data directly needed for dose metrics calculation, the other method for plan quality evaluation is visual evaluation. The aim is to obtain a uniformly distributed 50% and 25% isodoses around the target and also obtain a high dose fall-off from inside the GTV to the margin of PTV, without any significant high doses in the margin from the GTV to PTV. This fall-off allows the centering of high doses in the center of the GTV structure. In order to do so, the approached method in our departments is to optimize the plan on structures like internal GTV (internal margin of 2–3 mm from the GTV), a ring structure of the GTV (initial GTV from which we subtract the internal GTV), and a ring structure of the PTV (PTV from which we subtract the GTV). One of the limiting factors that constrains the steepness of the fall-off is the dose maximum allowed by the physician.

The statistical tools employed in this study were Spearman correlation analysis, simple T-testing, Anova, and multiple linear regression. We found the most adequate tool to use, in order to describe plan quality index behavior, to be linear regression as it can be used to model the relationship between multiple variables allowing us to estimate the separate contribution of each variable while others are held constant.

For a simple linear relationship, one can use the generic model [11] of the form:

(1) $$y=\beta_{0}+\beta_{1}x+\varepsilon ,$$

where y is the dependent or response variable, x is the independent or predictor variable, and ε is the error term, a statistical term representing random fluctuations, measurement errors, or the effect of outside factors within the model not explained by the x variables. As the response variable is often influenced by more than one predictor, the experimenter may add additional predictors with their own regression coefficients β.

The models may be linear with respect to β, but not necessarily in the x variables; therefore, our model—and a similar one for GM—can still be classified as a linear model.

(2) $$\hat{GI}=\beta_{0}+\beta_{1}\ln\left(TV\right)+\beta_{2}{\left[ln\left(TV\right)\right]}^{2}+\beta_{3}\left(\frac{Dose}{Fr}\right)+\beta_{4}MT+\varepsilon ,$$

where TV is target volume, dose/fraction is the physician-elected fractionation of the SRT plan, and MT is a flag type variable that describes whether or not the target was treated alone in a plan or together with multiple targets. The models aim to predict and explain the influence of our variables, helping guide planning decisions and set realistic expectations for achievable results. Of course, this relationship is subject to change with the advent of new technologies and novel techniques.

The study was conducted using data from patients treated between 2021 and 2025. In April 2025, preliminary approval was obtained from the ethics committee. Between April and October, anonymized dosimetric data were retrospectively processed, and in October 2025, final ethical approval was granted following the completion of the study and the presentation of the results, prior to submitting the manuscript to the journal.

The authors declare that no generative AI tools were used in data analysis or manuscript writing.

## 3. Results

Our study focuses on testing the correlation significance and computing Spearman correlation value for GI/GM and TV. The choice was to use TV and not equivalent sphere diameter (EQSD) due to the granularity of the data, as both showed strong statistical significance (*p* value of 9.01 × 10^−77^ for EQSD and 1.38 × 10^−76^ for TV) with regard to correlation to gradients (Spearman correlation of −0.908 for EQSD and −0.907 for TV).

We investigated various correlations between gradient, plan geometry, and dose metrics. There is a weak or moderate correlation between them, even though a strong statistical significance was shown. However, after running *t*-tests and generating several regression models trying to explain why geometry did not seem to affect our model, we realized our data are biased by our planning protocols.

Particular consideration was given to the number of couch kicks or positions in order to investigate the influence of plan geometry on gradient. However, a Spearman correlation between the number of couch positions and gradient revealed an insignificant (*p* = 0.76), very weak correlation (−0.02). An analysis of variance (Anova) between plans with three, four, five, and six couch positions also showed no statistical significance (*p* = 0.34) when looking at gradient index variances, further pointing towards a geometry bias in our dataset. We also tested whether doubling the number of arcs per couch position improved gradients; however, our data are biased, as all plans were originally created with sufficient or excess arcs and couch positions to avoid affecting the gradient. To be more specific, if the gradient of the plan was not acceptable initially, the plan was modified in geometry in order to fulfill the criteria established in our department. Therefore, with our current dataset we cannot evaluate meaningful changes based on plan geometry.

**Figure 3:**
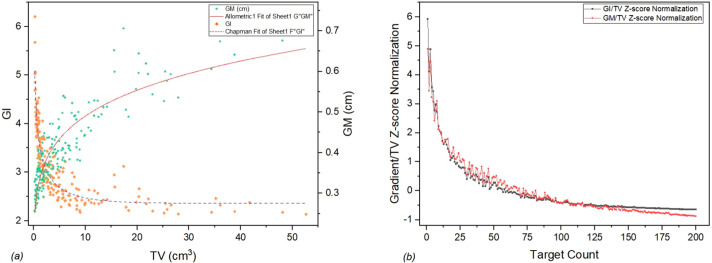
Clinical GI and GM dependance on TV (**a**) and renormalized gradients (**b**).

In an effort to better visualize the dependance of GI and GM depending on TV, while highlighting their differences, we have generated a graph of Standardized GI and GM using z-score normalization to account for the difference in units (Figure 3).

As previously discussed, two linear regression models were generated for gradient index and gradient measure as summarized Table 3 and Table 4.

In order to account for the exponentiality of the TV regressor in an easy-to-conceptualize way, we decided to change it to a base 2 logarithm when computing the first derivative, such that we may express the variability of our response variable (GI/GM) for every TV doubling.

(3) $$\frac{\partial \hat{GI}}{\partial ln(TV)}=\beta_{1}+2\beta_{2}ln(TV),$$

Rescaling to log (2) to express doubling using log change in base formula:

(4) $${log}_{2}\left(TV\right)=\frac{\ln\left(TV\right)}{ln2},$$

Rewrite predicted (fitted) GI function as:

(5) $$\hat{GI}=\beta_{0}+\left[\beta_{1}ln2\right]{log}_{2}\left(TV\right)+\left[\beta_{2}{\left(ln2\right)}^{2}\right]{\left[{log}_{2}(TV)\right]}^{2}+\beta_{3},$$

(6) $$\frac{\partial \hat{GI}}{\partial {log}_{2}(TV)}=\left[\beta_{1}ln2\right]+2\left[\beta_{2}{\left(ln2\right)}^{2}\right]{log}_{2}(TV),$$

Input our coefficients:

(7) $$\frac{\partial \hat{GI}}{\partial {log}_{2}(TV)}=-0.55156898+0.1152584{log}_{2}(TV),$$

For any given lesion, doubling its volume is predicted to change GI by $-0.55156898+0.1152584\times {log}_{2}(TV)$ . The behavior for a large range of TV is displayed in Table 5.

Similarly for any given lesion, doubling its volume is predicted to change GM by $0.024157\mathrm{cm}+0.013865\mathrm{cm}\times {log}_{2}(TV)$ . The behavior for a large range of TV is displayed in Table 6.

## 4. Discussion

The linear regression models presented in this study provide a quantitative, data-driven framework for stereotactic radiotherapy plan quality assurance in our clinic. By estimating the expected GI and GM for a given target volume and configuration, planners and physicists can rapidly identify plans that deviate from institutional protocols. It can become a useful tool for standardizing treatment plans across dosimetrists and physicists and set realistic expectations for achievable dose gradients at different target sizes.

Better understanding of the relationship between plan quality metrics, such as gradient, conformity and plan geometry would also provide a useful tool for standardizing treatment planning protocols across different centers. However, this would require a treatment planning system-generated water phantom in which you can test multiple geometries while keeping a constant TV, dose prescription, anatomical features, and target position relative to plan isocenter.

Several limitations of this study should be acknowledged. The analysis was retrospective and based on a single treatment technology (Varian TrueBeam), which may limit generalizability to other systems or planning algorithms. In addition, the models were derived from plans generated within only two clinical workflows, using our own internal optimization routines and external validation was not performed.

It can be seen from Figure 1 that target volumes larger than 20 cc are considered outliers. However, according to internal protocol, these kinds of large tumor volumes are also treated based on individual physician decisions with a larger number of fractions (e.g., five fractions). Such targets, while relatively uncommon, are still useful datapoints in determining the limitations of the protocol, technique, and technology observed in Figure 3a as a plateau.

New technologies (such as Varian Hyperarc) and planning techniques (Rapidarc Dynamic) open new possibilities of automated plan development and delivery as well as hybrid VMAT-IMRT delivery. The impact of these emergent technologies on plan quality indexes has not been approached by this study and remains a possible future direction of research [12].

Future research should focus on multi-institutional validation and comparison of regression parameters across planning systems and dose-calculation algorithms. This approach will be extended to other SRS/SRT non-cranial targets (lung, liver, and spine) in future research that is under development by our research team. For a more comprehensive model, future work should also focus on adapting this modeling approach to other SRS/SRT delivery systems to see if similar predictive relationships hold.

## 5. Conclusions

When evaluating small lesions, GI seems to be the more sensitive gradient evaluation metric for sub-cc SRT targets. Every doubling of target volume lowers GI sharply (−0.55 to −0.17). GI does exhibit an odd behavior within our model after 28 cc. We attribute this not to a true physical phenomenon, but rather to a modeling artifact resulting from the small number of cases in this upper volume range within our dataset. For lesions larger than 40 cc, the GI tends to have a constant behavior, without any significant increase nor decrease in value. The behavior of GI for larger than 20 cc volumes is influenced by the reduced number of data points in this region.

Treating multiple targets in the same plan seems to add an average of +0.19 to GI, independent of volume. Within our model, dose per fraction had no significant independent effect (*p* = 0.08). The model explains 85% of the variation in GI (adjusted R^2^ = 0.85).

When evaluating small lesions, GM seems to increase moderately (+0.024 cm to +0.07 cm). GM, according to our findings, displays a weaker sensitivity to volume than GI, making it perhaps a more useful tool when comparing GM across a wide volume spectrum of targets, though very large targets still show a penalty. Treating multiple targets in the same plan seems to add an average of +0.027 cm to GM, independent of volume. Dose per fraction had no significant effect (*p* = 0.1). The model explains 84% of GM variability. By verifying SRS/SRT plans using our model during the optimization process, the physics team can improve the dosimetric outcomes of the plans, with minimal time span. The generated models provide expected values of both gradient metrics for any target volume, offering a useful tool for assessing plan quality.

## Figures and Tables

**Table 1:** A summary of the investigated plan quality metrics.

Formulation	Parameter Description
${HI}_{RTOG}=\frac{I_{max}}{RI}$	$I_{max}$ = maximum dose to PTV RI = reference dose to PTV
${GI}_{PADDICK}=\frac{R_{50\%}}{V_{RI}}$	$R_{50\%}$ = volume of 50% isodose line $V_{RI}$ = volume of reference isodose line
$GM=\sqrt[{3}]{\frac{{3V}_{50\%Rx}}{4\pi }}-\sqrt[{3}]{\frac{{3V}_{Rx}}{4\pi }}$	$V_{50\%Rx}$ = volume receiving a dose equal to or greater than 50% prescription dose $V_{Rx}$ = volume receiving a dose equal to or greater than 100% prescription dose
${CI}_{PADDICK}=\frac{{TV}_{RI}^{2}}{TV\times V_{RI}}$	${TV}_{RI}$ = volume of target covered by reference isodose line $V_{RI}$ = volume of reference isodose line *TV* = target volume

HI = Homogeneity index; GI = Gradient index; GM = Gradient measure; CI = Conformity index.

**Table 2:** Relevant planning recorded variables.

Recorded Variables	Description
Prescribed Dose (Gy)	Dose prescription to 100% of target volume in Gray
Dmax (Gy)	Maximum dose expressed in Gray
Fr. Nr.	Number of prescribed fractions
Dose/Fr (Gy/fr)	Dose per fraction
Arc Nr.	Number of arcs in plan used to treat specified target
Couch Position Nr.	Number of couch positions including initial position
MT	A flag type variable that indicates whether the plan used to treat the referenced target contained multiple targets
Clinic	A flag type variable that indicates where the plan was calculated and treated
$\mathrm{TV}\;({\mathrm{cm}}^{3})$	Target volume
EQSD (cm)	Equivalent sphere diameter

**Table 3:** Gradient Index Regression summary.

GI Regression Statistics				
**Multiple R**	**R Square**	**Adjusted R Square**	**Standard Error**	**Observations**
0.924	0.854	0.851	0.286	200
	**Coefficients**	**Standard Error**	**t Stat**	***p*** **-value**
Intercept	3.674	0.0641	57.338	5.138 × 10^−124^
${\ln(TV)}^{2}$	0.119	0.0112	10.699	2.566 × 10^−21^
Dose/Fr	−0.00587	0.00330	−1.777	0.0770
ln(TV)	−0.795	0.0321	−24.767	3.967 × 10^−62^
MT	0.194	0.0463	4.195	4.141 × 10^−5^

**Table 4:** Gradient Measure Regression summary.

GM Regression Statistics				
Multiple R	R Square	Adjusted R Square	Standard Error	Observations
0.917	0.841	0.838	0.0400	200
	**Coefficients**	**Standard Error**	**t Stat**	***p*-value**
Intercept	0.33	0.00899	36.727	1.492 × 10^−89^
${\ln(TV)}^{2}$	0.0144	0.00157	9.174	6.612 × 10^−17^
Dose/Fr	0.000759	0.000463	−1.637	0.103
ln(TV)	0.0348	0.00451	7.731	5.524 × 10^−13^
MT	0.0271	0.00649	4.174	4.499 × 10^−5^

**Table 5:** GI behavior on TV.

**TV (cc)**	$\log_{2}(\mathrm{TV})$	**GI Change for the Next** **Doubling**	**Interpretation**
0.25–0.5	−2	−0.78	For very small lesions GI displays the most dramatic change indicating high-size sensitivity
0.5–1	−1	−0.67	
1–2	0	−0.55	GI drops by ~0.55 units. Small targets see the steepest improvement when they grow.
3–6	1.585	−0.37	Still a sizeable drop, but less significant
10–20	3.322	−0.17	Effect of getting larger is tapering off
28–56	4.807	~0	This is the turning point; GI is at its minimum.
40–80	5.322	+0.06	Beyond ~30 cc, further doubling starts to shift GI upward

**Table 6:** GM behavior on TV.

**TV (cc)**	$\log_{2}(\mathrm{TV})$	**GM Change for the Next** **Doubling**	**Interpretation**
0.25–0.5	−2	≈0 (−0.003)	In very low cc targets, GM is not a useful differentiator. In small cc targets, GM displays a nearly volume-neutral increase
0.5–1	−1	+0.010	
1–2	0	+0.024	GM increases by only 0.024 cm for small targets
3–6	1.585	+0.046	A slightly larger increase
10–20	3.322	+0.070	The per-doubling penalty grows—GM increases to 0.07 cm
28–56	4.807	+0.091	For TV > 30 cc, the rise in GM approaches ~0.1 cm for each doubling
40–80	5.322	+0.098	For very large targets, each doubling adds ~0.1 cm to GM

## Data Availability

Data are available only upon request due to ethical restrictions. The data presented in this study are available upon request from the main or corresponding author.

## Abbreviations

The following abbreviations are used in this manuscript:

GI	Gradient Index
GM	Gradient Measure
SRS	Stereotactic Radiosurgery
SRT	Stereotactic Radiation Therapy
TV	Target Volume
MT	Multiple Targets
HD-MLC	HD Multi-leaf Collimator
SGRT	Surface Guided Radiation Therapy
CT	Computed Tomography
RGSC	Respiratory Gating for Scanners
DIBH	Deep Inspiration Breath Hold
EEBH	End Expiration Breath Hold
HI	Homogeneity Index
CI	Conformity Index
AAA	Anisotropic Analytical Algorithm
EQSD	Equivalent Sphere Diameter
DVH	Dose Volume Histogram
PTV	Planning Target Volume
PRV	Planning Risk Volume
GTV	Gross Tumor Volume
SBRT	Stereotactic Body Radiation Therapy
